# Supplementary material for: Virus-like particle-based vaccines targeting the Anopheles mosquito salivary protein TRIO
Source: mSphere. 2025 Jan 29;10(2):e00798-24. doi: 10.1128/msphere.00798-24 (PMC11852919; doi:10.1128/msphere.00798-24)
Supplement: Figure S1 — Parasite liver burden. [file msphere.00798-24-s0001.docx]

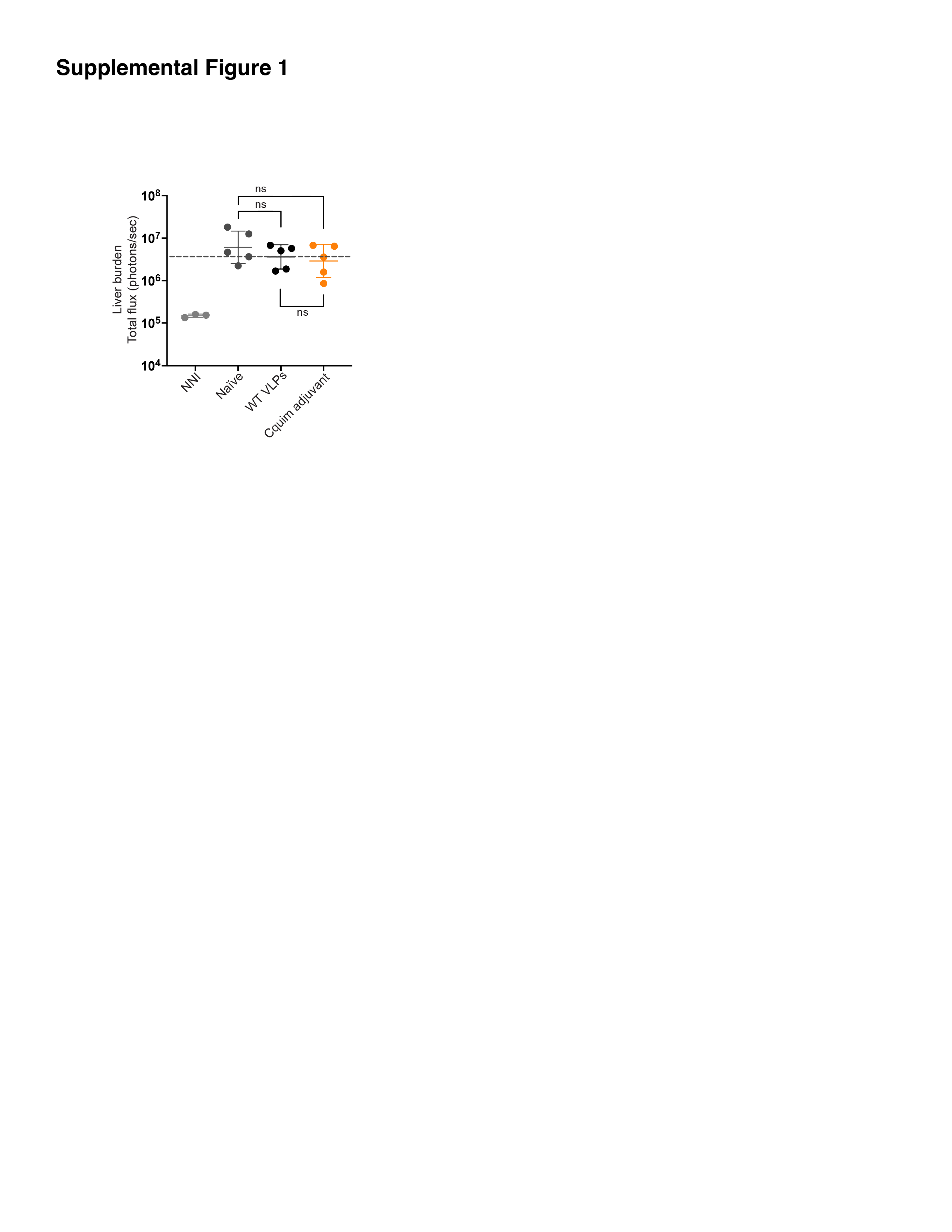


**Supplemental Figure 1.**

Female C57BL/6 mice (n=5) were immunized with 5µg WT VLPs or 2µg Cquim-MA adjuvant alone and boosted 4 and 8 weeks later. Mice were challenged with five Pb-PfCSP-Luc infected mosquitos. Parasite liver burden was measured by luminescence 42 hours after mosquito challenge. The grey dotted line marks the mean liver burden of mice immunized with WT VLPs. Background luminescence was determined using three uninfected mice (NNI, naïve non-infected). Data is reported as geometric mean ± geometric SD.
